# Evaluating the Impact of the Healthy Beverage Executive Order for City Agencies in Boston, Massachusetts, 2011–2013

**DOI:** 10.5888/pcd12.140549

**Published:** 2015-09-10

**Authors:** Angie L. Cradock, Erica L. Kenney, Anne McHugh, Lisa Conley, Rebecca S. Mozaffarian, Jennifer F. Reiner, Steven L. Gortmaker

**Affiliations:** Author Affiliations: Erica L. Kenney, Rebecca S. Mozaffarian, Jennifer F. Reiner, Steven L. Gortmaker, Department of Social and Behavioral Sciences, Harvard T. H. Chan School of Public Health, Boston, Massachusetts; Anne McHugh, Chronic Disease Prevention and Control, Boston Public Health Commission, Boston, Massachusetts; Lisa Conley, Intergovernmental Relations, Boston Public Health Commission, Boston, Massachusetts.

## Abstract

**Introduction:**

Intake of sugar-sweetened beverages (SSBs) is associated with negative health effects. Access to healthy beverages may be promoted by policies such as the Healthy Beverage Executive Order (HBEO) established by former Boston mayor Thomas M. Menino, which directed city departments to eliminate the sale of SSBs on city property. Implementation consisted of “traffic-light signage” and educational materials at point of purchase. This study evaluates the impact of the HBEO on changes in beverage availability.

**Methods:**

Researchers collected data on price, brand, and size of beverages for sale in spring 2011 (899 beverage slots) and for sale in spring 2013, two years after HBEO implementation (836 beverage slots) at access points (n = 31) at city agency locations in Boston. Nutrient data, including calories and sugar content, from manufacturer websites were used to determine HBEO beverage traffic-light classification category. We used paired *t* tests to examine change in average calories and sugar content of beverages and the proportion of beverages by traffic-light classification at access points before and after HBEO implementation.

**Results:**

Average beverage sugar grams and calories at access points decreased (sugar, −13.1 g; calories, −48.6 kcal; p<.001) following the implementation of the HBEO. The average proportion of high-sugar (“red”) beverages available per access point declined (−27.8%, p<.001). Beverage prices did not change over time. City agencies were significantly more likely to sell only low-sugar beverages after the HBEO was implemented (OR = 4.88; 95% CI, 1.49–16.0).

**Discussion:**

Policies such as the HBEO can promote community-wide changes that make healthier beverage options more accessible on city-owned properties.

## Introduction

Intake of sugar-sweetened beverages (SSBs) is associated with increased risk of type 2 diabetes (1), coronary heart disease (2,3), and excess weight gain (4). Decreasing SSB consumption could reduce the prevalence of obesity and obesity-related diseases (5). Although overall SSB consumption has declined over the last decade (6), low-income Americans of all ages are more likely to be heavy SSB consumers than their higher-income counterparts (7). On average, Americans consume approximately 150 kcals per day from SSBs, the equivalent of just over one 12-ounce serving per day (6). Recently, nutrition standards for school lunch and breakfast programs established by the Healthy Hunger-Free Kids Act of 2010 helped to ensure the availability of healthy choices at school (8). Policies specifying the provision of only healthy beverage options in school settings have been linked with decreased overall beverage consumption among students (9). However, SSBs are widely available in other community locations (10–14), indicating that other setting-specific policies could promote wider access to healthy beverage options.

Many voluntary community and organizational initiatives include healthy beverage campaigns, which set nutrition standards for beverages sold or provided in various settings (15). Pilot initiatives to increase access to healthy beverages in vending machines by inserting nutrition standards into vending contracts in 3 Delaware state agency buildings were successful several weeks after initiation (13). However, in recreational settings and health services organizations, issuing voluntary, recommended nutrition guidelines did not result in consistently healthy beverage and food offerings (16,17), particularly when nutrition standards were not incorporated into the contracting processes (18). Outcome evaluations of healthy beverage promotion policies are limited.

In April 2011, Boston’s former mayor, Thomas M. Menino, issued the Healthy Beverage Executive Order (HBEO), which went into effect in October 2011 (19). This executive order directed city departments to eliminate the sale of SSBs on city property and to adhere to the City of Boston’s HBEO standards in vending machines and city-managed food or beverage services programs. The HBEO standards were developed by the Boston Public Health Commission in response to the HBEO and outlined the requirements for beverages that could be sold (19). Calorically sweetened beverages, including some energy drinks, sports drinks, sweetened tea, and coffee drinks, were allowed if they contained less than or equal to 1 gram of sugar per fluid ounce. These standards also addressed portion size for certain categories of beverages (eg, milk, milk substitutes) and product mix (ie, diet or other noncalorically sweetened beverages must make up no more than one-third of total offerings). The objective of this study was to evaluate whether access to healthy beverages had increased in Boston city agencies 2 years after the HBEO was issued.

## Methods

### Study design

This policy evaluation uses a pre–post natural experimental design (20) to evaluate the impact of the HBEO on changes in healthy beverage availability in Boston city agencies. Beverage access data were collected by trained data collectors before (March–September 2011) and after (March–November 2013) the HBEO was issued. Additional data were collected in local recreation sites not subject to the HBEO in July–August 2011 and June–July 2013.

The HBEO directed Boston City agencies to eliminate SSBs from city-funded events and vending machines and from cafés or cafeterias on city property. It also restricted purchase of SSBs with city funds and prohibited certain types of industry marketing on city property (eg, banners, vending machine graphics) that promoted products that did not qualify for sale under HBEO standards (19). The HBEO also directed the formation of the Healthy Options Coordinating Committee (HOCC). The HOCC included representatives of relevant city departments and, under the leadership of the Boston Public Health Commission, coordinated implementation of the HBEO, conducted an inventory of beverage points of purchase and existing beverage contracts and policies, and provided communication and educational materials about the HBEO standards (19). These communication and education materials were included in the healthy beverage toolkit (21). The toolkit contained information about beverage standards and resources for implementing the HBEO requirements in Boston city agencies and other worksite settings in Boston. The toolkit included point-of-decision consumer educational materials that used a traffic-light system to identify categories of beverages (ie, red designates “drink rarely, if at all,” yellow designates “drink occasionally,” and green designates “drink plenty” or “healthy choice.”) (Box). The Boston Public Health Commission also provided city agencies with brochures, posters, and other promotional and education materials that used these traffic-light identifiers (21). The HOCC met 5 times over 6 months. It created sample standard contract language regarding the healthy beverage standards that agencies could incorporate easily in city contracts. Additional technical assistance was provided regarding specific venues that were subject to the HBEO. Other opportunities included free workshops focused on the implementation of nutrition policy change, including such topics as working with contractors and vendors, technical assistance on legal issues, procurement policies, and special dietary needs.

**Box. Boston Public Health Commission’s Point-of-Purchase Traffic-Light Classification System for Beverages^a^**

**Table:** 

Beverage Color Classification	Criteria	Examples
Red: drink rarely, if at all	Over 12 g sugar per 12 oz	•Regular soda •Energy drinks (regular) •Sports drinks (regular) •Pre-sweetened coffee and tea drinks •Juice drinks with added sugar •Whole or 2% milk
Yellow: drink occasionally	6 g to 12 g of sugar per 12 oz or contains artificial sweeteners	•Diet soda •Diet iced tea •100% fruit juice (in small portions) •Low-calorie sports drinks •Sweetened soymilk (in small portions) •Flavored 1% milk (in small portions) •Other low-sugar drinks •Energy drinks (artificially sweetened and/or containing ≤1 g sugar/oz) •Sports drinks (artificially sweetened and/or containing ≤1 g sugar/oz)
Green: drink plenty	0 to 5g of sugar per 12oz	•Water •Seltzer water •1% or skim milk (in small portions) •Unsweetened soymilk (in small portions)

^a^ Boston Public Health Commission. Healthy Beverage Toolkit: Boston Public Health Commission; 2011. http://bphc.org/whatwedo/healthy-eating-active-living/healthy-beverages/Documents/HealthyBeverageToolkitFinal.pdf.

### Sample

**Beverage access points in Boston city agencies.** To assess changes in access to healthier beverages, we generated a list of city properties (n = 115) that served as access points (vending machines, cafés, or cafeterias where beverages could be purchased) in Boston city agencies. Schools were excluded because their beverage policy prohibited the sale of SSBs (9). Individual City of Boston parks were evaluated as part of a separate survey described below. We identified agency contacts and scheduled appointments to tour each facility. Fire departments (n = 36 properties) and police departments (n = 12 properties) agreed to participate in implementing the policy but declined to participate in the assessment protocol. Of the remaining 67 properties, 27 were public libraries, 37 were community centers, and 3 were administrative buildings. Data collectors visited these 67 city properties at baseline in 2011 (before the implementation of the HBEO). Of these, 28 city properties were identified, representing 45 beverage access points. At follow-up in 2013, seven properties (6 community centers and 1 library) representing 4 access points had closed, and data collectors visited the remaining 60 properties. In addition, 4 properties had removed 10 vending machines representing 10 access points. This yielded a total reduction of 14 access points from baseline, leaving 31 beverage access points. The 14 access points that closed were not included in the longitudinal analysis. Therefore, the longitudinal analysis included 22 properties representing 31 access points that were present at both baseline and follow-up (Figure).

**Figure:**
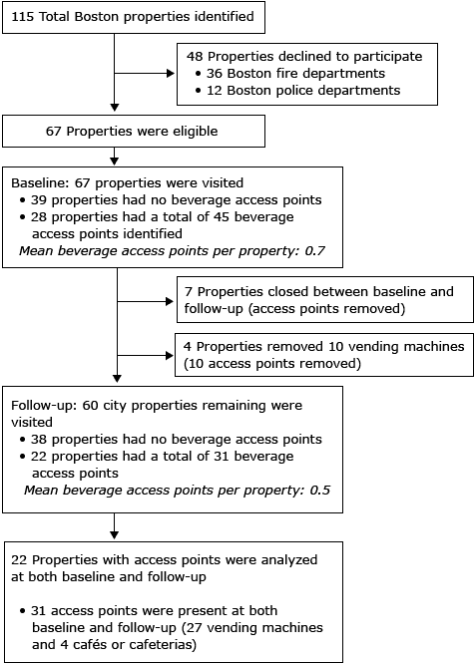
Boston city properties that participated in the evaluation of the Healthy Beverage Executive Order, 2011–2013.

**Beverage access points in Boston parks and recreational facilities.** From June through July 2011, data were collected on water access and beverages for sale at parks and recreational facilities operated by the Boston Parks Department (a Boston city agency) and by the Massachusetts Department of Conservation and Recreation (DCR). From July through August 2013, researchers revisited these parks and recreational facilities. The accessibility of seasonal recreational facilities and beverage access points differed at baseline and follow up. Longitudinal analyses therefore include 5 locations with beverages available at both baseline and follow up, consisting of 4 Boston Parks Department locations and 1 DCR location. Only Boston Parks were subject to the HBEO; therefore, the 1 DCR recreational facility served as a control location.

### Measures

**Beverages at access points on city properties.** Research assistants located beverage access points on each city property and used a standard protocol to record the location within the agency building (eg, floor, building number, nearest office) and the brand, type, flavor, size, and price of each beverage available. Digital photographs of access points were used to document brand marketing and to identify beverage slot facings (ie, selection slots or spaces on the shelf facing the consumer) in vending machines and in cafeteria or café coolers and refrigerated cases. Researchers collected data on the price, brand, and size of beverages for 899 beverage slots in spring 2011 and 836 beverage slots in spring 2013, 2 years after the HBEO was issued.

**Beverages at access points in parks and recreational facilities.** Data collection in parks and recreation facilities employed an abbreviated protocol recording the brand, type, flavor, size, and price of each unique beverage (without photos). Researchers collected data on the price, brand, and size of 51 beverages at baseline in 2011 and 93 beverages at follow-up in 2013.

**Beverage nutrient information.** Researchers collected nutrient and ingredient information for each beverage from manufacturers’ websites or by contacting manufacturers. When brand-specific information was not available (eg, brewed coffee, tea), standard nutrient information by beverage type was obtained from the US Department of Agriculture nutrient database (22). The nutrient variables included total energy (in kcals), sugar (in grams) per serving and where applicable, noncaloric sweetener type (artificial or natural noncaloric).

### Analysis

**Traffic light beverage and access point classifications.** Researchers classified beverages and beverage slot facings found at baseline and follow-up according to the traffic light categorization developed by the Boston Public Health Commission (21) and standards outlined in the HBEO standards (19). Within each access point, researchers calculated key outcomes consisting of the proportion of green, yellow, and red beverages available and the average beverage calories (kcal), sugars (g), and price (USD) by using the slot facings data. Researchers also classified access points by other relevant criteria: 1) contained no red beverages, 2) contained a beverage mix of no more than 1/3 artificially sweetened yellow beverages, and 3) contained a mix of beverages where at least 2/3 of the available beverages were green or yellow and were not artificially sweetened, and 4) marketing no red beverages. To enable comparison, all prices were reported in 2011 dollars (23).

The primary outcomes were the changes in the average proportion of beverages classified as red, yellow, or green at each access point. Secondary outcomes were changes in the average calories, sugar content, and price by access point. Paired *t* tests assessed differences in the primary and secondary outcomes at each access point, before and after the HBEO was issued. We used logistic regression to determine the change in the likelihood of selling any red beverages at baseline versus follow-up. We also compared outcomes from the access points that were removed at follow up with the longitudinal sample by using *t* tests to evaluate potential selection bias resulting from loss to follow-up. For data available from parks and recreational facilities, we calculated the proportion of beverages available by traffic light classification and the average calories, sugar content, and price. Significance was set at *P* < .05 and analyses were conducted by using SAS statistical software version 9.3 (SAS Institute, Inc).

## Results

Twenty-two Boston city properties contained 31 beverage access points (27 vending machines and 4 cafés or cafeterias) present at both baseline and follow-up (Figure). These points contained 899 beverage slot facings before implementation of the HBEO and 836 after implementation. The mix of beverages changed at access points after the implementation of the HBEO (Table 1). At baseline, access points contained an average of 26.8% green, 32.7% yellow, and 40.5% red beverage facings. There was a small, nonsignificant change in the percentage of green beverages facings at follow-up, but yellow beverage facings increased by 26.1 (<.001) and red beverage facings decreased by 27.8 (*P* < .001) after the implementation of the HBEO. When stratified by access point type (ie, vending versus café or cafeteria), access to green beverages in cafeterias or cafes increased by 9.6% (*P* = .03), whereas yellow beverage access increased in vending machines by 28.4% (*P* < .001). Red beverage access in vending machines decreased by 28.9% (*P* < .001) and in cafés or cafeterias by 20.4% (*P* = .02).

**Table 1:** Beverages Available on Boston City Properties by Access Points (N = 31) and Traffic-Light Classification System^a^ Before and After Issuance of the Healthy Beverages Executive Order, March–September 2011 Through March–November 2013

Access Points	Green Beverages, % (SD)^b^				Yellow Beverages, % (SD)^b^				Red Beverages, %, (SD)^b^			
	Baseline	Follow-up	Average Change	*P *Value^b^	Baseline	Follow-up	Average Change	*P *Value^b^	Baseline	Follow-up	Average Change	*P *Value^b^
Total access points (N = 31)	26.8 (21.3)	28.5 (20.2)	1.7 (19.6)	.64	32.7 (21.8)	58.9 (23.3)	26.1 (24.7)	<.001	40.5 (24.4)	12.7 (18.1)	−27.8 (24.5)	<.001
Vending machines (N = 27)	28.8 (22.1)	29.3 (21.5)	0.5 (20.7)	.91	33.1 (23.3)	61.5 (23.1)	28.4 (25.4)	<.001	38.1 (25.2)	9.2 (16.2)	−28.9 (25.9)	<.001
Cafeteria or café (N = 4)	13.1 (1.5)	22.7 (5.1)	9.6 (4.7)	.03	30.4 (6.2)	41.2 (18.2)	10.9 (12.3)	.17	56.5 (5.7)	36.1 (13.9)	−20.4 (8.3)	.02

Abbreviation: SD, standard deviation.

a Green beverages = drink plenty (water, seltzer water, skim or 1% milk); yellow beverages = drink occasionally (diet soda, low-calorie or low-sugar drinks, or 100% juice); red beverages = drink rarely, if at all (regular sodas, energy or sports drinks, or fruit drinks).

b Totals may differ slightly because of rounding.

c *P* values are the results of paired *t* tests.

The average calories per beverage sold within access points decreased between baseline and follow-up by 48.6 kcal, from 88.1 kcal to 39.5 kcal, *P* < .001 (Table 2). Baseline calories of beverages from cafes or cafeterias (136.7 kcal) were higher than calories of beverages in vending machines (80.9 kcal). The average sugar content of beverages from either source also decreased between baseline and follow-up by 13.1 g, from 22.8 g to 9.7 g (*P* < .001). Sugar content was higher at baseline for beverages sold in cafés or cafeterias (32.8 g) than for beverages sold from vending machines (21.3g). Beverage prices did not differ between baseline and follow-up (*P* = .96).

**Table 2:** Nutritional Quality and Price of Beverages Available on Boston City Properties by Access Points (N = 31) and Traffic-Light Classification System^a^ Before and After Issuance of the Healthy Beverages Executive Order, March–September 2011 through March–November 2013

Access Point	Green Beverages, Mean (SD)		Yellow Beverages, Mean (SD)		Red Beverages, Mean (SD)		Total Beverages, Mean (SD)			
	Baseline	Follow-up	Baseline	Follow-up	Baseline	Follow-up	Baseline	Follow-up	Average Change	*P* Value^b^
**Average calories per beverage (kcal)**	1.7 (6.4)	0.9 (3.4)	41.5 (39.1)	28.6 (26.9)	184.3 (41.5)	174.2 (54.7)	88.1 (47.7)	39.5 (38.4)	−48.6 (44.9)	<.001
Vending machine	0 (0)	0 (0)	38.2 (41.1)	23.6 (19.2)	180.2 (44.0)	171.3 (61.1)	80.9 (46.8)	32.4 (33.9)	−48.6 (47.5)	<.001
Cafeteria or café	12.1 (14.0)	7.2 (7.6)	60.9 (16.2)	61.1 (48.2)	206.7 (4.2)	182.8 (33.7)	136.7 (12.0)	87.5 (35.9)	−49.2 (24.6)	.03
**Average sugar content per beverage (g)**	0.2 (0.8)	0.1 (0.4)	9.5 (8.8)	6.7 (5.8)	48.4 (11.3)	44.0 (12.9)	22.8 (12.4)	9.7 (9.4)	−13.1 (12.0)	<.001
Average sugar content, vending machine beverage	0.0 (0.1)	0 (0)	9.1 (9.3)	5.8 (4.6)	47.8 (12.2)	44.5 (14.5)	21.3 (12.6)	8.2 (8.8)	−13.1 (12.8)	<.001
Average sugar content, cafeteria or café beverage	1.5 (1.7)	0.8 (0.9)	12.0 (4.3)	12.8 (9.8)	51.4 (3.0)	42.6 (7.2)	32.8 (4.2)	19.7 (7.7)	−13.1 (3.8)	.006
**Average price per beverage, $^c^**	1.25 (0.24)	1.25 (0.26)	1.38 (0.29)	1.37 (0.25)	1.39 (0.29)	1.47 (0.32)	1.34 (0.26)	1.34 (0.25)	0 (0.16)	.96
Average price per beverage, $^c^, vending machine	1.23 (0.20	1.22 (0.25)	1.32 (0.26)	1.32 (0.19)	1.32 (0.24)	1.34 (0.17)	1.29 (0.21)	1.29 (0.19)	0 (0.15)	.95
Average price per beverage, $^c^, cafeteria or café	1.41 (0.38)	1.47 (0.29)	1.76 (0.20)	1.72 (0.34)	1.76 (0.28)	1.86 (0.36)	1.72 (0.27)	1.71 (0.33)	0 (0.25)	.99

Abbreviation: SD, standard deviation.

a Green beverages = drink plenty (water, seltzer water, skim or 1% milk); yellow beverages = drink occasionally (diet soda, low-calorie or low-sugar drinks, or 100% juice); red beverages = drink rarely, if at all (regular sodas, energy or sports drinks, or fruit drinks).

b *P* values are the results of paired *t* tests.

c Price data for 2009 baseline values are inflation-adjusted to 2011 to allow for direct comparison.

At baseline, 5 access points did not sell any beverages designated “red”. At follow-up, 15 access points had eliminated all red beverages, and access points were significantly more likely to offer no red beverages (OR = 4.88; 95% CI, 1.49–16.0, *P* = .009) than at baseline. There was no change in the number of access points offering one-third or fewer beverages with artificial sweeteners designated “yellow” (N = 17). The number of access points meeting the HBEO marketing criteria (ie, marketing only healthy beverages) was the same at baseline and follow-up (28 access points, 90.3%). The access points that had been closed or removed at follow-up had higher-priced beverages at baseline than those access points available at both time points ($1.29 vs $1.07, *P* = .005).

In comparisons of recreational facilities, an average of 61.1% of 45 beverage offerings at 4 access points in City of Boston properties were classified as red in 2011, and 30.4% of 81 beverage offerings were classified as red at follow-up in 2013. In the single DCR site, beverages designated red constituted 83.3% of 6 offerings at baseline and 83.3% of 12 offerings at follow-up. The site-level average calories per beverage offering at Boston recreation sites was 123.3 kcal (SD, 44.9) at baseline and 83.3 kcal (SD, 64.9) at follow-up, whereas the average calories per beverage offered at access points in the DCR site were 140.2 kcal at baseline and 122.1 kcal at follow-up.

## Discussion

This study suggests that policies supporting access to healthy beverages on city-owned properties can make healthier beverage options more accessible to city residents and employees at those locations. After the HBEO was issued, the availability of healthier beverage options increased significantly in vending machines, cafeterias, and cafés on city properties. City agencies were also significantly more likely to offer only healthier beverages for sale after the executive order was issued. We observed declines in the sugar content and calories in beverages available for sale at city properties alongside the 28% average decline in the proportion of high-sugar (red) beverages available for sale at city properties in Boston with no change in the price. We found no change in availability of healthy beverage choices in the DCR comparison recreation site in Boston that was not subject to the HBEO during the same time period.

This work supports the findings of a growing number of studies that suggest that policies and healthful vending initiatives can affect local access to healthy options in community settings (13,18,24). However, specific policy content may affect implementation, sustainability, and impact. Although Boston properties were more likely to be free of less healthy (ie, red) beverages after the HBEO restricted their sale, not all properties met the executive order’s standard. Implementation differed by access point; it was lower among cafeterias and cafés on city properties than at vending machine points. In prior studies, binding procurement contract provisions, a limited choice of available options from contracted vendors that meet nutrition standards, and concerns about competitive sales environments or loss in profit were barriers to full implementation of nutrition guideline initiatives (17,25). To facilitate implementation and sustainability in Boston, future contracts could be negotiated with inclusion of HBEO criteria. In prior studies, the timely inclusion of nutrition standards in procurement contracts was noted as a factor in the successful implementation of a policy promoting healthy beverage options (18). Additionally, policies requiring 100% healthy beverages may facilitate compliance because of their focus on promoting healthy choices better than a predetermined mix of options (eg, 50% healthy) that require ongoing monitoring for product mix compliance.

This study had limitations. We lacked data on procurement policies, consumer impact, and beverage sales, which limited our assessment of effects on product-specific or category-specific procurement and sales. However, studies of similar labeling and education programs alongside policies promoting greater access to healthy options have demonstrated increased purchasing of healthy options. For example, in prior studies in hospital cafeterias, educational labeling programs were associated with significantly increased purchasing of healthy options. When accompanied by increased accessibility of healthy choices, purchase of healthy options again increased and purchases of less healthy options declined (26,27). At follow-up in Boston cafés and cafeterias, the average price of beverages designated green ($1.47) was substantively lower than that of the less healthy options available ($1.86). Differential pricing of healthy beverages below that of less-healthy beverages can promote increased purchases of healthier beverages in cafeteria settings (28). Additionally, upgrading vending machines to healthy options only has been associated with increased average monthly per-machine sales (24).

Other limitations are that we assessed beverage availability in these settings but did not collect data among control site locations in other business vending or cafeteria locations. However, we did not observe increases in the availability of healthy beverage choices in the DCR recreational facility we visited that was not subject to the HBEO. Additionally, some agencies declined to participate in the beverage access assessment protocol, so we lacked data on these locations.

Following the mayor’s announcement of the HBEO, 10 Boston-area hospitals also opted to make healthy beverages conveniently accessible to their employees and patrons. As city officials review and revise the HBEO standards, they should consider the facilitators of implementing healthy beverage policies, such as contractual agreements with vendors that incorporate new standards, limiting exemptions, and providing additional technical assistance and capacity for compliance-monitoring and feedback (17,18). Monitoring efforts could use existing frameworks for both foods and beverages sold in publicly funded institutions (29).

Community-wide access to healthier beverage alternatives can be promoted by policies such as the HBEO, which directed Boston city properties to eliminate the sale of SSBs. Two years after the executive order was issued, healthier beverage options were more accessible to city residents and employees in vending machines and in cafeterias and cafés on city properties. Additionally, city agencies were more likely to offer only healthier beverages for sale after the executive order was issued with no increase in beverage prices. During the same time period, increased access to healthy options was not found in a DCR facility in Boston that was not subject to the policy.
